# Retraction Note: Circular RNA ATXN7 promotes the development of gastric cancer through sponging miR-4319 and regulating ENTPD4

**DOI:** 10.1186/s12935-025-03733-x

**Published:** 2025-03-13

**Authors:** Zhen Zhang, Honglei Wu, Zhaosheng Chen, Guangchun Li, Bin Liu

**Affiliations:** Department of Gastroenterology, The Second Hospital of Shandong University, No. 247 Beiyuan Street, Jinan, 250033 Shandong China

**Retraction Note: Cancer Cell Int (2020) 20:25** 10.1186/s12935-020-1106-5

The Editors-in-Chief have retracted this article. After publication, concerns were raised regarding highly similar images between Fig. 5C well 3 in this article and Fig. 1f well 1 in the authors' earlier study [1]. Further checks by the publisher have found that three of the cell lines used in this article were contaminated with HeLa cervical cancer cells, which was confirmed by STR analysis.

The authors have been unable to share the full raw data upon request. The Editors-in-Chief therefore no longer have confidence in the presented data.

Honglei Wu has not explicitly stated whether they agree to this retraction. None of the other authors have responded to any correspondence from the editor or publisher about this retraction.
